# Spatial encoding of a traumatic virtual reality scene reduces intrusive memories

**DOI:** 10.1093/scan/nsag037

**Published:** 2026-05-21

**Authors:** Thomas Meyer, John King, Pauline Dibbets, Melanie Dari, Neil Burgess, Richard Benning, Jacco Ronner, Nexhmedin Morina, Chris R Brewin

**Affiliations:** Clinical, Educational and Health Psychology, University College London, Gower Street, London, WC1E 6AE, United Kingdom; Institute of Psychology, University of Münster, Fliednerstraße 21, Münster, 48149, Germany; Clinical, Educational and Health Psychology, University College London, Gower Street, London, WC1E 6AE, United Kingdom; Clinical Psychological Science, Maastricht University, Universiteitssingel 40, 6229 ER Maastricht, The Netherlands; Institute of Psychology, University of Münster, Fliednerstraße 21, Münster, 48149, Germany; Institute of Cognitive Neuroscience, University College London, 17-19 Queen Square, London, WC1N 3AZ, United Kingdom; Clinical Psychological Science, Maastricht University, Universiteitssingel 40, 6229 ER Maastricht, The Netherlands; Clinical Psychological Science, Maastricht University, Universiteitssingel 40, 6229 ER Maastricht, The Netherlands; Institute of Psychology, University of Münster, Fliednerstraße 21, Münster, 48149, Germany; Clinical, Educational and Health Psychology, University College London, Gower Street, London, WC1E 6AE, United Kingdom

**Keywords:** intrusive memories, virtual reality, dual representation theory, spatial memory training, post-traumatic stress disorder

## Abstract

We tested whether enhanced allocentric spatial memory of a distressing scene can attenuate intrusive memories. Using virtual reality (VR), we developed an immersive trauma analog to measure and manipulate spatial memory. Study 1 (*N *= 92) established the paradigm’s validity in eliciting intrusions and assessing spatial memory for scene-embedded objects. Results showed that spatial memory was better for more distressing objects and when participants were probed from the original encoding viewpoint, but memory performance did not differentiate egocentric from allocentric strategies or relate to intrusions during a 15-minute interval. Critically, Study 2 (*N *= 120) showed that targeted spatial memory training on scene contents, administered after viewing the traumatic scene, significantly reduced intrusions, as assessed by a 3-day intrusion diary and a retrospective self-report assessment, compared to a perceptual control. These findings support our main hypothesis, suggesting that therapeutic approaches targeting intrusions may benefit from enhancing memory for the spatial layout of traumatic events. Further investigation is needed to determine the specific role of allocentric memory, as opposed to general spatial memory, in this process.

In Western countries, 50%–80% of adults experience traumatic events such as terrorist attacks or life-threatening accidents, with many developing recurrent intrusive trauma memories and other symptoms of post-traumatic stress disorder (PTSD; Darves‐Bornoz *et al.* 2008, De Vries and Olff 2009, Morina *et al.* 2014, Brewin *et al.* 2025). Despite their clinical relevance and societal impact, early interventions are still limited and the mechanisms driving intrusive memories are still poorly understood (Bryant 2021). Therefore, theory-driven research is urgently needed to push toward novel intervention strategies.

Information processing theories suggest intrusive memories arise from perceptual trauma memories that are overly accessible and insufficiently processed. Individuals with PTSD may overly rely on egocentric memory representations (i.e. remembering the traumatic event from the first-person perspective), lacking a more elaborated spatial representation (Brewin *et al.* 2010, Bisby *et al.* 2020). Accordingly, individuals who primarily encode their own egocentric perspective and fail to contextualize the event in time and space should be more vulnerable to intrusive re-experiencing. This may be mitigated by hippocampal-dependent allocentric representations that bind the locations of environmental features relative to each other (Brewin *et al.* 2010). Therefore, strengthening allocentric spatial representations relative to predominantly egocentric representations should reduce intrusions.

Indirectly supporting these assumptions, intrusive memories and PTSD symptoms have been linked to dysfunction in the hippocampal area, crucial for visuospatial processing and the contextual embedding of memories (Francati *et al.* 2007, Gilbertson *et al.* 2007). Trauma analog studies in healthy participants have associated intrusive memories with contextual and spatial configuration learning deficits (Meyer *et al.* 2013, 2019a, Xu *et al.* 2024). Conversely, acute cortisol stress responses and negative affective stimuli have been linked to attenuated learning of spatial configurations (Meyer *et al.* 2019b) and associative binding in memory (Bisby *et al.* 2016, 2020). More specifically, allocentric memory formation inversely correlates with PTSD symptoms (Bisby *et al.* 2010, Smith *et al.* 2015, Sierk *et al.* 2019). Since allocentric spatial learning can be viewed as a tractable form of hippocampal associative binding, which is thought to support coherent episodic memories (Bisby and Burgess 2017, Zlomuzica *et al.* 2018), these findings highlight the potential for diagnostic and therapeutic interventions targeting allocentric memory formation.

Together, converging evidence is consistent with the revised dual-representation theory of PTSD (DRT; Brewin *et al.* 2010, Bisby *et al.* 2020), which hypothesizes that intrusions stem from relatively weak allocentric/contextual trauma representations. This view also aligns with separable medial temporal lobe systems for item/perceptual detail versus context/relational encoding (Davachi 2006, Ritchey *et al.* 2015). Yet, no published study has experimentally validated this causal claim. Therefore, the present research experimentally manipulates allocentric memory formation within an analog traumatic experience and examines its impact on subsequent intrusive memories.

## Present studies

In Study 1, we developed and validated a paradigm capable of provoking intrusive memories in healthy participants while assessing allocentric spatial memory of distressing scene elements. We used a laboratory analog trauma model (Dibbets and Schulte-Ostermann 2015, Cuperus *et al.* 2016, Dibbets 2020) in virtual reality (VR) to create an immersive scene depicting a passenger airplane crash, with aversive and non-trauma related neutral objects. VR allows full control over viewpoints and scene elements, making it an ideal tool to measure and train spatial abilities (Rose *et al.* 2000, Bohil *et al.* 2011).

Study 2 aimed to manipulate spatial memory experimentally. Participants were randomly assigned to either a spatial location memory training condition or a perceptual control condition, and we investigated group differences in spatial memory and intrusion frequency.

## Transparency and openness

### Preregistration

Design and analyses of Study 1 and 2 were not preregistered. However, they were specified in a third-party grant proposal (https://doi.org/10.3030/749206). These studies represent initial efforts to investigate the causal role of spatial memory in intrusions using novel measures and manipulations, setting the stage for future confirmatory research (Mayo 2018, Scheel *et al.* 2021).

### Data availability

All anonymized data, analysis code and syntax for reproducing the presented findings, full instruction transcripts, description of materials, and sample items of the aversive VR scenario are available on OSF via this link (see the Supplementary Material). Below, we report how we determined our sample size, all data exclusions, all manipulations, and all measures relevant to this study.

## Study 1

### Design and hypotheses

**Figure 1:**
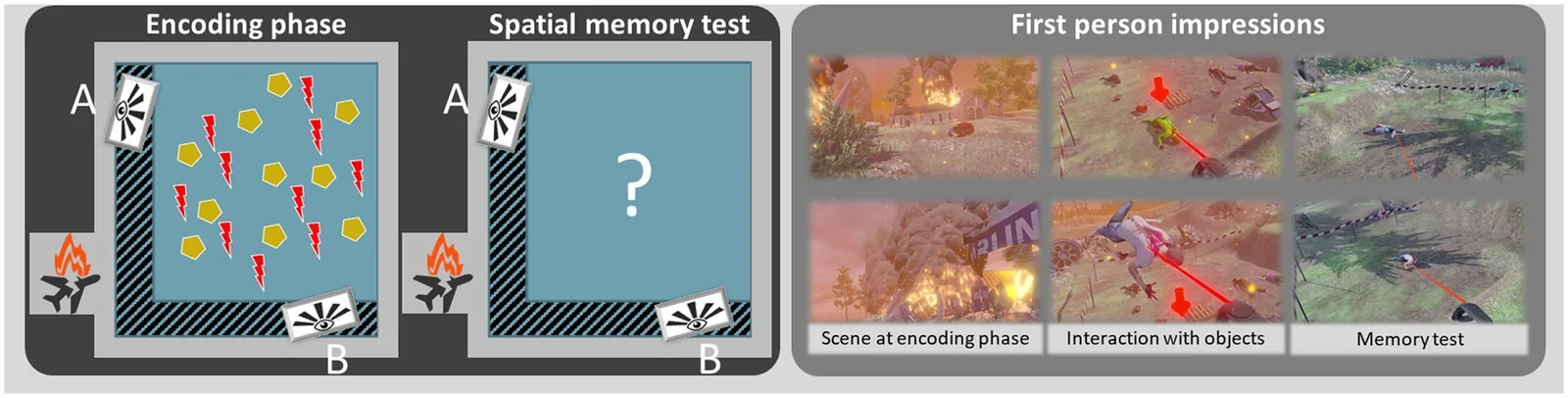
Schematic overview of the scene layout and first-person impressions. *Note*: Following brief exploration in the dark area, participants were placed at one of two fixed viewpoints (A or B) differing by 140° in orientation. During encoding, the scene depicted aversive objects (e.g., body parts) and neutral items (e.g., a fence post), illustrated here by lightning- and pentagon-shaped placeholders, that were evenly distributed, excluding corners. The landscape included distinct orientation points in all directions (e.g., trees, hills, and a farmhouse). During the memory test, the scene was cleared, and location memory was tested for one object at a time. These tasks were developed using Unity3D (Unity Technology, USA) and presented on an HTC Vive Pro headset (HTC Corporation, USA).

Study 1 employed a within-person design to assess the relationship between spatial memory for scene elements, individual differences in spatial abilities, and intrusive memories. This initial study aimed to validate the VR scenario, depicted in Fig. 1, which simulates a passenger airplane crash site with 16 objects: 8 aversive and 8 neutral, located within a 10 m × 10 m area. Participants viewed the scene from one of two fixed viewpoints, and memory for object locations was later tested both from the original and a shifted perspective. We assessed the occurrence of intrusive memories during a 15-min break to estimate the effectiveness of the paradigm to elicit intrusions (in the literature, intrusions are generally measured as they occur in everyday life over 3–7 days). The Four Mountains Test (4MT; Hartley *et al.* 2007), measuring topographical memory, and the Town Square task (TST; King *et al.* 2002, King *et al.* 2004), measuring location memory from different viewpoints, were used as convergent measures gauging allocentric memory abilities (for detailed descriptions, see Supplementary Material: link).

We expected better spatial memory from the original viewpoint (H1a) and for aversive objects (H1b), reflecting emotional arousal effects (Yonelinas and Ritchey 2015, Talmi *et al.* 2019). We further expected individual differences in allocentric spatial abilities to predict better spatial memory generally (H2a), with positive correlations specifically from the shifted viewpoint (H2b). Positive associations between viewpoint-dependence in spatial memory and intrusive memories (H3) were also addressed, albeit through exploratory analyses, as intrusive memories were only assessed during a brief 15-min period.

### Method

#### Participants

Ninety-two healthy participants (72 women, 20 men, age *M *= 20.9, *SD *= 3.8, range: 18–34) were recruited via the online platform SONA (https://www.sona-systems.com) at University College London (UCL) from 10/2018 to 02/2019. Two additional participants withdrew. Of the final sample, 89.1% were students, 9.8% employed. Inclusion criteria: aged 18–35, normal or corrected vision. Exclusions (all based on self-report): pregnancy, psychological/psychiatric treatment, neurological disorders, psychoactive medication, high regular alcohol or drugs use, fear of blood, and past traumatic experiences. Compensation was 20 GBP or course credits. Study 1 had UCL Ethics Committee approval (Project ID: DSD/2014/001).

Sample size determination targeted moderate-to-large effects (e.g. *r* = .30–.50), using bivariate correlation as a pragmatic proxy due to the inherent complexity of conducting power analysis directly for Linear Mixed Models (LMMs). With *N *= 85 power was 80% to detect *r* = .30 with a two-sided 5% alpha, achieving actual power of 83.5%.

#### Procedure

Candidates who passed the online self-screening attended a single 120-minute laboratory session. For sample description, we included the Depression, Anxiety and Stress Scales (DASS-21; Lovibond and Lovibond 1995), the State-Trait Anxiety Inventory—Trait subscale (Spielberger 1983), and the Santa Barbara Sense-of-Direction Scale (SBSoD; Hegarty *et al.* 2002). Details and exploratory analyses involving these scales can be found on the OSF. Following this series of questionnaires, participants completed the TST and 4MT, prior to VR briefing, orientation, and aversive VR scenario. Affective responses were tracked using the Positive and Negative Affect Schedule (PANAS; Watson *et al.* 1988), consisting of two 10-item subscales (score range: 10–50) for positive affect (PA; αs > .911) and negative affect (NA; αs > .764) before/after the scenario. After the aversive scenario, participants underwent a 15-min rest, followed by retrospective assessment of uncued intrusions and administration of the VR spatial memory test. Detailed descriptions of each task can be found via this link (see Supplementary Material).

#### Statistical analysis

Analyses used IBM SPSS (version 31) and RStudio (version 2026.01.0) for LMM of Euclidean distance errors (m) in the VR spatial memory task. Robust models with HC3 correction (White 1980) and Satterthwaite’s approximation were applied when homoscedasticity assumptions were violated. A null model determined appropriate random effects, retaining only effects with ≥5% explained variance; participant identity met this criterion, explaining 11.89%. Models addressing H1 included Viewpoint, Valence, and interactions. Models addressing H2 included the convergent variables tapping into spatial abilities (4MT accuracy; TST same-view and shifted-view accuracies), as well as the interaction terms with Viewpoint (computed following mean-centering) as predictors. H3 was addressed using a Pearson correlation between intrusion frequency during the 15 min break and a VR-task derived viewpoint-dependence (VD) score for aversive objects (mean distance error_shifted_ – mean distance error_original_). Higher VD scores indicate greater viewpoint dependence.

### Results

#### Affective responses

Paired *t*-test showed that PANAS-NA increased from 13.40 (*SD *= 4.09) to 18.24 (*SD *= 6.64), *M_Diff_* = 4.84 (*SD *= 5.93), *P* < .001, *d *= 0.82. PANAS-PA also increased from 21.62 (*SD *= 9.70) to 24.26 (*SD *= 9.12), *M_Diff_* = 2.64 (*SD *= 9.17), *P* = .007, *d *= 0.29. Unexpectedly, PANAS-PA increased; compared with Study 2 this appears driven by low baseline PA in Study 1 (Study 2: *M* = 30.47, *SD* = 5.87). This is likely because the PANAS was administered after the attentionally demanding TST and 4MT.

#### Uncued intrusive memories

Intrusion frequency averaged 1.86 (*SD *= 1.81, range: 0–8), slightly below “occasionally.” Mental images (*M *= 2.18, *SD *= 2.18, range: 0–8) exceeded verbal thoughts (*M *= 0.97, *SD *= 1.59, range: 0–7), but were less frequent than deliberate thoughts (*M *= 3.79, *SD *= 2.54, range: 0–9), with all *P*s < .001 (Bonferroni). Intrusions were rated as slightly to moderately distressing (*M *= 2.14, *SD *= 2.31, range: 0–8) and vivid (*M *= 3.66, *SD *= 3.13, range: 0–10), with slight physical reactions (*M *= 1.57; *SD *= 2.24; range: 0–8). In text responses, 63 participants (68.5%) described intrusion content, while 29 (31.5%) indicated “none.” Most descriptions (see Supplementary Material: link) referenced scenario objects (e.g., “*the broken leg*,” “*burnt dead body*”), other audio-visual details (“*screams*”), or details from the briefing video (“*purpose of the flight*”).

#### Spatial memory (H1a-b)

LMMs on distance errors demonstrated main effects for Viewpoint (original, shifted), *t*(8737) = 2.584, *P* = .010, and Valence (aversive, neutral), *t*(8737) = 6.511, *P* < .001, supporting H1a-b. The interaction term was not significant, *P* = .267. Model diagnostics indicated variance inflation factors (VIFs) of 10 for the main effects and 19 for the interaction. Removing the interaction reduced VIFs to 1. Robust analysis confirmed the Viewpoint effect (estimate = 0.14, *SE *= 0.03, *t*(91) = 4.74, *P* < .001, *d *= -0.10), with smaller distance errors for the original (EMM = 1.93, 95% CI: 1.82, 2.05) than for the shifted viewpoint (EMM = 2.07, 95% CI: 1.96, 2.19), and Valence effect (estimate = 0.51, *SE *= 0.06, *t*(91) = 8.34, *P* < .001, *d *=* −*0.37), with smaller distance errors for aversive objects (EMM = 1.75, 95% CI: 1.63, 1.86) than for neutral objects (EMM = 2.26, 95% CI: 2.14, 2.37). A descriptive visualization of the H1a-b effects is provided in Fig. S1 (see online supplementary material, OSF).

#### Association with spatial ability (H2a-b)

The LMM on distance errors with 4MT-accuracy indicated a 4MT-accuracy × Viewpoint interaction (as predicted by H2b) close to significance, *t*(8547) = −1.787, *P *= .074. In a refined robust model excluding the interaction, 4MT-accuracy had a significant negative main effect (as predicted by H2a; standardized coefficient = −0.16, 95% CI: −0.29, −0.02), *t*(20.5) = −2.25, *P* = .036. Separate robust models for each viewpoint indicated a slightly smaller negative association of 4MT-accuracy with distance errors at the original (standardized coefficient = −0.13, 95% CI: −0.25, −0.01) compared to the shifted viewpoint (standardized coefficient = −0.18, 95% CI: −0.34, −0.03). The LMM with TST accuracies showed no interactions, *P*s > .173, but a main effect for shifted-view TST accuracy, *t*(283.7) = −2.326, *P* = .021. In a standardized robust model excluding interactions, shifted-view TST accuracy no longer met the threshold of significance (standardized coefficient = −0.20, 95% CI: −0.40, 0.00), *t*(26.2) = −1.988, *P* = .057.

#### Association with intrusive memories (H3)

The VD score for aversive objects did not correlate with intrusive memories (*P* = .850). Details, as well as correlations between scores on the 4MT and TST with intrusive memories, are summarized in Table 1.

**Table 1:** Reported intrusion characteristics and correlations with affect and spatial abilities.

Outcome	*M*	*SD*	Pearson correlation (*N *= 92) [95% CI]				
			NA_change_	PA_change_	TST score^a^	4MT^a^	VD score
**Any intrusion**	1.86	1.81	.40*** [.21; .56]	−.26* [−.44; −.06]	−.07 [−.28; .14]	−.23* [−.42; −.02]	.02 [−.19; .22]
**Mental images**	2.18	2.18	.38*** [.19; .54]	−.19 [−.38; .02]	−.07 [−.27; .14]	−.27** [−.45; −.07]	.03 [−.18; .23]
**Verbal thoughts**	0.97	1.59	.00 [−.21; .20]	−.08 [−.28; .13]	−.11 [−.31; .10]	−.06 [−.26; .15]	−.07 [−.27; .13]
**Distress**	2.14	2.31	.49*** [.31; .63]	−.26* [−.44; −.06]	−.03 [−.23; .18]	−.12 [−.32; .09]	−.12 [−.32; .09]
**Vividness**	3.66	3.13	.41*** [.22; .57]	−.15 [−.34; .06]	−.15 [−.34; .06]	−.23* [−.42; −.03]	.05 [−.16; .25]
**Physical reaction**	1.57	2.24	.28** [.03; .46]	.00 [−.21; .20]	−.01 [−.22; .20]	−.05 [−.26; .16]	−.05 [−.25; .16]
**Deliberate thought**	3.79	2.54	.38*** [.19; .54]	−.13 [−.33; .07]	−.13 [−.33; .08]	.08 [−.13; .28]	.02 [−.19; .22]

*Note*. NA = Negative Affect subscale of the Positive and Negative Affect Schedule (PANAS); PA = Positive Affect subscale of the PANAS; TST score = Town Square Task allocentric memory score; 4MT = 4 Mountains Task accuracy; VD = viewpoint-dependence score (VR-task derived).

a *N *= 90.

* *P* < .05.

** *P* < .01.

*** *P* < .001.

### Study 1: Discussion

Our VR paradigm successfully elicited intrusive memories during a post-encoding break, though intrusion frequency was relatively low, likely due to the short measurement period without reminder cues. Intrusion levels can be expected to be higher over the usual post-exposure period of 3–7 days in other trauma-analog studies. Positive correlations between intrusions and negative affect (see Table 1) align with studies using the trauma-film paradigm (Clark *et al.* 2015, 2016). Notably, negative correlations with 4MT accuracy, but not TST scores, partly align with prior findings on spatial abilities and intrusions (Meyer *et al.* 2013, Sierk *et al.* 2019). Consistent with DRT, these correlations were specific to intrusive mental images, not verbal thoughts (Brewin *et al.* 2010; Hagenaars *et al.* 2010).

Spatial memory of the aversive scenario was enhanced from the original versus the shifted viewpoint (H1a), consistent with earlier studies (King *et al.* 2002, 2004), and more accurate for aversive versus neutral objects (H1b), replicating the emotional arousal effect (Yonelinas and Ritchey 2015, Talmi *et al.* 2019). The validity of the VR spatial memory paradigm was further substantiated by its correlation with the 4MT, a measure of topographical memory (H2a), though this correlation was not specific to memory performance from the shifted viewpoint (H2a). Despite similar trends, no robust correlation with the TST was observed.

Contrary to H3, we found no evidence that intrusion levels are associated with viewpoint differences in VR spatial memory, despite the negative correlation between 4MT accuracy and intrusions. Possible explanations include masking of associations by the memory-enhancing effect of negative affect (H1b); insensitivity of the spatial memory paradigm to distinguish egocentric versus allocentric strategies (see H2b); and the low number of intrusions during the brief assessment period.

In summary, Study 1 confirms our VR paradigm’s effectiveness in eliciting intrusions and capturing spatial scene memory. However, mixed findings regarding theory-derived predictions highlight the need for experimental manipulation of spatial memory to investigate its role in intrusive memories, as addressed in Study 2.

## Study 2

### Design and hypotheses

**Figure 2:**
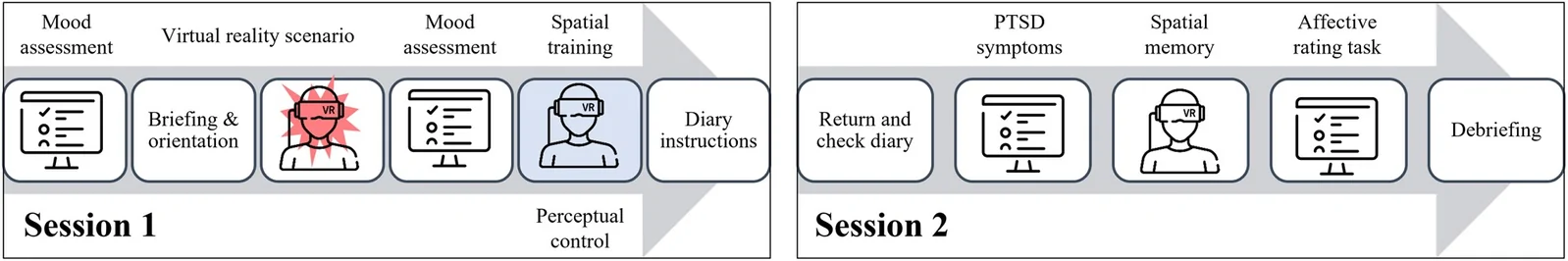
Timeline of Study 2.

Study 2 experimentally manipulated spatial memory of VR scene objects *after* exposure to the aversive scene, to test its effect on reducing intrusive memories. Spatial training was compared to a perceptual control condition, which maintained engagement with scene elements without focusing on spatial locations. The study timeline is depicted in Fig. 2. Our primary hypothesis was that spatial training would be followed by fewer diary-recorded intrusions than the perceptual control condition (H1). Additionally, we explored correlations between spatial memory for aversive objects and intrusions (H2).

### Method

#### Participants

One-hundred and twenty participants (81 women, 36 men, 3 gender diverse; mean age 22.9, *SD *= 3.0, range: 19–32) completed this study from 02/2023 to 06/2024. One additional participant withdrew and was replaced. Of the final sample, 96.7% were students and 3.3% were employed. Inclusion and exclusion criteria matched Study 1. Compensation was 16€ or course credit. Study 2 was approved by University of Münster’s Ethics Committee (Project ID: 2023-04-ThM).

A priori power analysis, accounting for moderate effect sizes (*d *= 0.50; cf. Hedge’s *g* = 0.59 for manipulations of visuospatial processing during trauma exposure; Siehl *et al.*, submitted; also see Varma *et al.* 2024), determined 50 participants per condition for 80% power with a one-sided alpha of 0.05. An additional 20% increase was set to accommodate potentially smaller VR effects, yielding 80% power for an effect of *d *= 0.456, or alternatively, 85.9% power for *d *= 0.50.

#### Methodological changes

Several changes were implemented from Study 1 to enhance measurement precision and reliability. Intrusions were measured more thoroughly using a standard 3-day intrusion diary and using the Impact of Event Scale–revised (IES-R; Maercker and Schützwohl 1998), providing strong convergent validity for intrusion assessment (Singh *et al.* 2023). In VR, all participants viewed the aversive encoding phase from the same perspective to minimize variability and to enhance our ability to examine individual differences (Consequently, the effect size of the Viewpoint main effect, which is not a focus of this study, should be interpreted with caution, as it partly reflects absolute viewpoint differences, rather than only the difference between original and shifted perspectives). The number of neutral objects in the scenario and memory tasks was reduced to four, keeping eight aversive objects, to reduce overall set size and enhance memory performance (Cowan 2001, Bennett *et al.* 2009). This was primarily motivated by the task’s difficulty and comparatively low accuracy in Study 1, with the aim of increasing sensitivity to individual differences and training effects.

#### Spatial memory training

**Figure 3:**
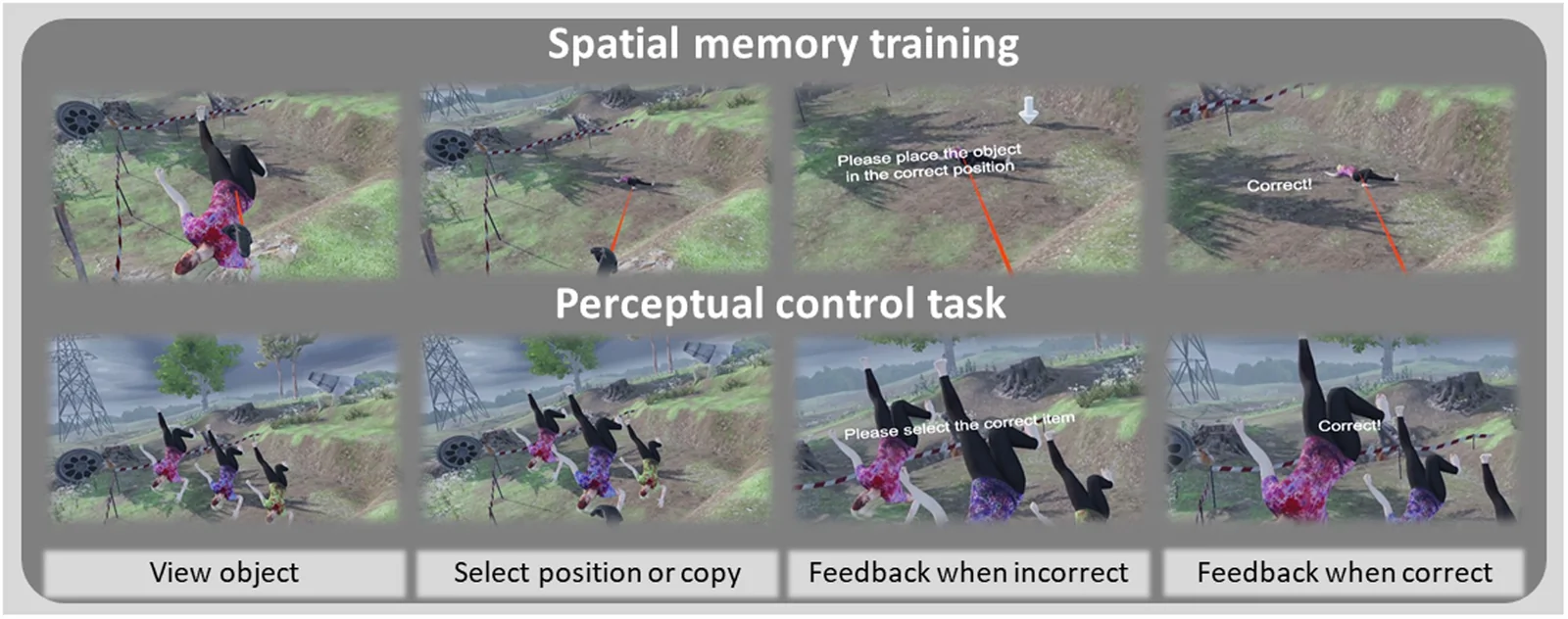
First person view impressions from the spatial memory training and perceptual control task.

Participants in the spatial training condition engaged in a VR task to enhance spatial memory immediately post-encoding. Standing at the original encoding viewpoint, participants sequentially positioned each object to its remembered location (see Fig. 3). Using the controller, they adjusted positions and confirmed them. Upon response, immediate feedback followed: the word “*Correct!*” was displayed for 1 s for proximity to the correct location, and the object remained visible for another 2 s before the next trial began. If the chosen location was incorrect, the feedback “*Please place the object in the correct position*” was displayed for 1 s with a guiding arrow above the correct location. Trials continued until each object was placed correctly three times.

The training focused solely on the original viewpoint for several reasons. First, even from the original viewpoint, the training was expected to enhance memory at least partly through allocentric strategies, allowing participants to construct a mental map that supports flexible recall from any viewpoint. Second, Study 1 found no reliable difference between the two viewpoints in their association with other spatial memory tasks. Third, shifting viewpoints during the spatial memory task would make it difficult to construct an equivalent control condition, since such a shift would alter scene perception and possibly foster allocentric processing while highlighting distressing details (e.g. of the plane wreck) not previously seen.

#### Perceptual control task

To match the spatial training’s duration and engagement with the objects, participants performed a perceptual decision task without spatial memory. On each trial, participants identified the original object from a lineup with two color-differing foils, displayed floating in the air at the same distance used in the spatial memory task. Using the controller, participants selected the original and confirmed their choice. Immediate feedback was given as in the spatial training task: “Correct!” or “Please select the correct object.” Trial number and sequence mirrored the spatial training condition.

#### Intrusive memories

Participants were extensively briefed on the definition of intrusive memories as sudden and unwanted recollections in the form of mental images, verbal thoughts, or both. They were provided with a 3-day structured pen-and-paper diary (Holmes *et al.* 2009, Meyer *et al.* 2019a) to record each intrusion and its modalities as soon as it occurred, or the absence of intrusions at least twice daily. For each memory, they then recorded content and triggering events for experimenter verification and rated the associated distress and vividness on 11-point scales (0 = *not at all*; 10 = *extremely*). Intrusion frequencies (total and by modality) were logarithm transformed (ln[1 + # intrusions]) for analysis, but non-transformed descriptive statistics are presented for readability. Distress and vividness scores were averaged across memories, with zero entered if no intrusion had occurred.

The revised Impact of Event Scale (IES-R; Maercker and Schützwohl 1998) requires respondents to indicate the frequency of stress-related symptoms on four-point scales scored non-linearly (0 = *not at all*; 5 = *often*). The Intrusion Symptoms subscale (7 items, α = .83) was of particular theoretical interest, while the total score (22 items, α = .83) served as an additional measure of overall analog PTSD symptoms.

#### Procedure

Participants attended two 45-minute laboratory sessions, three days apart. They gave informed consent and completed baseline questionnaires; For sample description, we included the DASS-21 (Nilges and Essau 2015); The Spontaneous Use of Imagery Scale (SUIS; Nelis *et al.* 2014) and the Brooding subscale of the Response Style Questionnaire (RSQ; Nolen-Hoeksema and Morrow 1991) were included to explore whether individual differences in imagery and/or rumination would moderate the experimental effects. Details and results of these analyses can be found on the OSF (see Supplementary Material: link). The PANAS was administered pre- and post-encoding (PA αs>= .81; NA αs>= .78). Post-encoding, they engaged in either spatial memory training or the perceptual control task, based on random assignment. They completed the intrusion diary over three days and returned it in session 2. The experimenter checked diaries for completeness and readability. Participants completed the IES-R, and then underwent the VR spatial memory test and affective ratings. Finally, participants were debriefed and compensated.

#### Statistical analysis

Data were analyzed using SPSS and RStudio, in line with Study 1. We conducted independent samples *t*-tests on diary intrusions and the IES-R scores. Associations between intrusion outcomes and task-derived viewpoint-dependence (VD) scores (as in Study 1) were examined using linear regression models including Condition, VD, and their interaction. LMMs explored spatial memory data, incorporating experimental condition as a between-subjects factor. A null model determined appropriate random effects, retaining only effects with ≥5% explained variance; participant identity met this criterion, explaining 25.81%. Alpha was set at 0.05 (two-sided) for all tests, except for H1, where alpha was set at 0.05 (one-sided) due to the confirmatory and directional nature of this hypothesis.

### Results

#### Affective responses

A mixed ANOVA revealed an increase in NA from pre- to post VR scenario (prior to the experimental manipulation) from 13.64 (*SD *= 3.61) to 19.34 (*SD *= 5.23) points, *M_Diff_* = 5.70 (*SD *= 5.90; 95%CI: 4.63, 6.77), *F*(1,118) = 113.8, *P* < .001, η_p_^2^ = .491, as well as a decrease in PANAS-PA from 30.47 (*SD *= 5.87) to 25.58 (*SD *= 5.71) points, *M_Diff_* = −4.89 (*SD *= 5.84; 95%CI: −5.95, −3.84), *F*(1,118) = 84.7, *P* < .001, η_p_^2^ = .418. There were no unintended main or interaction effects involving Condition prior to the manipulation, all *P*s >= .095, η_p_^2^ <= .02.

#### Manipulation check: spatial memory

**Figure 4:**
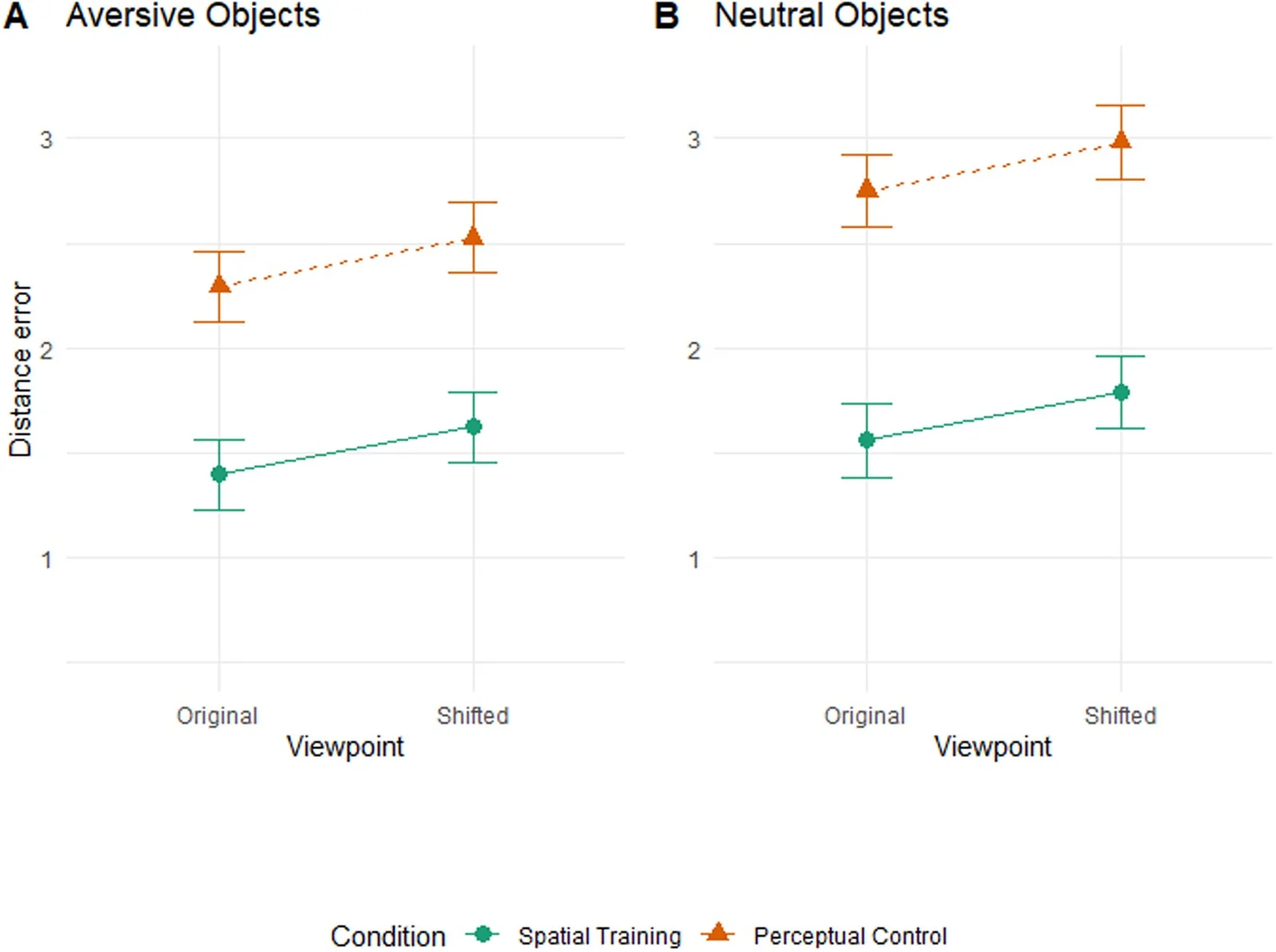
Distance errors in the VR spatial memory task for aversive objects (a) and neutral objects (b). *Note*: Although spatial training was administered from the original encoding viewpoint only, the delayed spatial memory test included both original- and shifted-viewpoint trials. The substantial effects of experimental conditions validate the spatial memory training, with learning effects lasting several days. However, note that training and testing were conducted on the same items, precluding an inference of a general improvement in spatial memory. Error bars represent 95% confidence intervals.

An LMM included Condition (spatial training, perceptual control), Viewpoint (original, shifted), Valence (aversive, neutral), and their interactions on distance errors. Significant main effects emerged for Condition (estimate = −1.23, *SE *= 0.13, *t*(164) = −9.72, *P* < .001), Viewpoint (estimate = 0.25, *SE *= 0.06, *t*(8515) = 4.31, *P* < .001), and Valence (estimate = −0.41, *SE *= 0.05, *t*(8515) = −7.79, *P* < .001). The interaction between Condition and Valence was significant (estimate = 0.29, *SE *= 0.06, *t*(8515) = 4.79, *P* < .001), whereas interactions involving Viewpoint were not (all *P*s > .12). Refined robust models with HC3 correction excluding nonsignificant interaction terms confirmed the significant main effects of Condition (estimate = −1.19, *SE *= 0.15, *t*(118) = −7.76, *P* < .001, *d *= 0.78), Viewpoint (estimate = 0.23, *SE *= 0.03, *t*(119) = 8.37, *P* < .001, *d *= 0.17), and Valence (estimate = −0.46, *SE *= 0.14, *t*(59) = −3.24, *P* = .002, *d *= −0.23), whereas the Condition × Valence interaction was now reduced and not significant at trend-level (*P* = .068). Exploratory simple effects analyses indicate that the spatial training condition may have had a slightly larger effect within neutral valence trials (estimate = −1.19, *SE *= 0.12, *P* < .001) than within aversive trials (estimate = −0.90, *SE *= 0.12, *P* < .001). Figure 4 depicts the estimated distance errors per condition.

#### Intrusive memories (H1)

As can be seen in Table 2, the primary finding of this study is that intrusion levels were lower in the spatial training condition than in the perceptual control condition, both in terms of intrusive memories recorded in the diary and in terms of retrospectively assessed intrusion symptoms (IES-R). Distress associated with intrusions was low to moderate in both groups, with lower distress reported in the spatial training condition. Exploratory analyses presented in the supplementary materials (see Supplementary Material: link) indicate that the experimental effects on intrusion symptoms remained statistically significant when controlling for individual differences in imagery and rumination tendencies.

**Table 2:** Group differences in the intrusion-related outcome variables (*N *= 120).

		Condition			Group statistics		
Measure	Subscale	Spatial training	Perceptual control	*M_diff_* [95% CI]	*d*	*t*	*P*
**#intrusions^a^**	Any type	2.70 (2.35)	4.13 (3.49)	−1.43 [−2.51; −0.36]	−0.49	−2.71	.004^b^
	Images	2.23 (1.91)	3.43 (2.83)	−1.20 [−2.07; −0.33]	−0.49	−2.69	.008
	Thoughts	1.38 (1.91)	2.27 (2.48)	−0.88 [−1.68; −0.08]	−0.45	−2.46	.016
**Distress**		2.03 (1.98)	2.89 (2.11)	−0.86 [−1.60; −0.12]	−0.42	−2.30	.012
**Vividness**		5.18 (3.13)	5.98 (2.30)	−0.80 [−1.80; −0.19]	−0.29	−1.60	.113^c^
**IES-R**	Intrusions	5.87 (6.14)	8.47 (5.26)	−2.60 [−4.67; −0.53]	−0.46	−2.49	.007^b^
	Total	13.1 (10.9)	18.1 (10.4)	−4.95 [−8.80; −1.10]	−0.47	−2.55	.012

*Note*. IES-R = Impact of Event Scale.

a Non-transformed intrusion means and CIs are presented for readability while the *t*-tests were based on log-transformed intrusion counts adjusting the typical right-skewed distribution.

b One-sided *P*-value, as these tests directly address directional Hypothesis 1a. All other *P*-values are two-sided.

c Equal variances not assumed, based on a significant Levene’s test.

#### Association with intrusive memories (H2)

VD scores for aversive objects did not predict intrusions or moderate the training effect: Condition × VD score interactions were non-significant for diary intrusions and IES-R intrusions (*p*s>=.261), and VD scores showed no reliable main effects (*p*s≥=.087), whereas the main effect of Condition remained significant (*p*s<=.015).

## General discussion

Building on Study 1, Study 2 confirmed our primary hypothesis that enhancing spatial memory for scene-embedded items reduces intrusion frequency compared to a perceptual control condition. This effect occurred robustly across measures, including a 3-day diary and retrospective self-report of PTSD symptoms. Exploratory analyses (see Supplementary Material: link) confirmed these outcomes when controlling for rumination and imagery tendencies. Consistent with Study 1’s finding that topographical memory accuracy (4MT) is associated with fewer image-based intrusions, these findings align with DRT predictions (Brewin *et al.* 2010, Bisby *et al.* 2020), indicating that enhancing spatial memory may be a viable intervention strategy for minimizing post-traumatic intrusive memories.

Our approach of *enhancing* visuospatial memory contrasts with earlier studies focused on disrupting memory (re-)consolidation via visuospatial tasks, like playing Tetris following trauma memory reactivation. These studies assume that visuospatial tasks compete for cognitive resources, thereby degrading, weakening, or blurring sensory elements of traumatic memories (Holmes *et al.* 2009, Iyadurai *et al.* 2018). At first glance our approach seems incompatible with these strategies, yet it achieves similar benefits.

A compelling explanation lies in DRT’s (Brewin *et al.* 2010) distinction between sensation-based and contextual memories. The theory suggests that intrusions can be reduced either by interfering with egocentric, sensation-based memory representations, as is achieved with trauma-unrelated visuospatial tasks (Holmes *et al.* 2009), or by strengthening allocentric contextual representations. Within this broader distinction, the perceptual control task likely engaged item-based perceptual-detail processing, whereas spatial training more directly recruited hippocampal-area based contextual (allocentric) encoding, providing a plausible neural account of the training-related intrusion reduction (Davachi 2006, Ritchey *et al.* 2015). Our findings align with other evidence that deliberately enhancing memory after viewing a trauma film reduces intrusions (Krans *et al.* 2009; Hørlyck *et al.* 2019; Xu *et al.* 2024), and that a spatial-schematic processing style may prevent PTSD symptoms in trauma-exposed individuals (Yeung *et al.* 2025). Our approach thus represents an alternative mechanism for reducing intrusions within the framework of DRT.

Alternative accounts of our results include that shifting focus onto spatial configurations during training may divert attention away from emotional content, potentially engaging working memory as hypothesized in Eye-Movement Desensitization and Reprocessing (EMDR; van Veen *et al.* 2020). This focus may also facilitate adoption of an observer vantage point, a strategy hypothesized to increase avoidance and reduce the emotional intensity of aversive memories (Kenny and Bryant 2007). In addition, spatial training may provide a sense of control, beneficial for trauma processing (Ehlers and Clark 2000). These divergent explanations warrant further investigation in future studies that may examine control, engagement, and attentional effects during spatial training.

### Clinical implications

Our findings suggest novel treatment strategies. Common traumatic events could be integrated in VR scenes with spatial learning tasks, including severe traffic accidents (Dibbets 2020), interpersonal violence (Dibbets and Schulte-Ostermann 2015, Loucks *et al.* 2025), or warzone experiences (Rizzo *et al.* 2009). Clinical translation faces challenges, including acceptance from both therapists and patients (e.g. van Meggelen *et al.* 2022). Alternative spatial interventions, like imagining the traumatic event from different perspectives (Kaur *et al.* 2016), or using idiosyncratic reminder cues instead of traumatic scene elements (Meyer *et al.* 2022), may offer practical solutions. Future studies should further explore these avenues to inform the development of novel intervention strategies.

From a clinical perspective, an important yet unresolved question is whether the effectiveness of a spatial memory training depends on the emotional valence of scene elements. In our study, the training had a substantial and consistent effect (*d *= 0.78) on spatial memory, independent of valence. Future studies should clarify whether training of neutral objects alone could yield beneficial effects on intrusions, as this could pave the way for effective interventions without requiring exposure to the most aversive details of a traumatic scene. Conversely, it is possible that inclusion of the most aversive scene elements is essential for reducing intrusive memories. Supporting this view, a study on imagery rescripting found that including the most aversive details of a traumatic film during intervention was beneficial for reducing subsequent intrusions (Dibbets and Arntz 2016). Hence, future research should explore these possibilities to inform potential clinical applications.

### Limitations

Several limitations warrant discussion. Although spatial memory training appeared effective, it remains possible that the perceptual control condition unintentionally increased intrusions compared to a baseline with no intervention. Moreover, our design cannot conclusively confirm allocentric memory as crucial. Study 1 correlations showed that allocentric abilities are linked with better spatial memory in VR, though this was not discernible between viewpoints. This limits the interpretability of correlational findings with intrusive memories. It is possible that repeated testing from original and shifted viewpoints in VR may let participants initially use egocentric memory before engaging in more allocentric strategies. Consequently, participants with deficient allocentric encoding may have formed allocentric memory during testing, suggesting that future modifications may improve diagnostic precision. Notably, intrusion measurement differed across studies (Study 1: immediate 15‑min post-encoding assessment; Study 2: 3‑day diary/IES‑R), limiting direct comparability and likely contributing to the low intrusion frequency and null correlational findings in Study 1. Finally, we used an analog trauma with healthy samples. Future studies should investigate these effects in trauma-exposed individuals with clinically relevant symptoms.

### Conclusions

This research suggests that trauma-focused spatial memory enhancement could reduce intrusive memories. By linking reduced intrusions to improved spatial/contextual memory, our findings tentatively support accounts in which hippocampal-area (allocentric/contextual) processing counterbalances predominantly egocentric, perceptual-detail representations implicated in PTSD, consistent with DRT and the Posterior-Medial/Anterior-Temporal (PMAT) framework (Brewin *et al.* 2010, Ritchey *et al.* 2015). While further investigation into mechanisms is needed, our findings highlight the potential of theory-driven approaches grounded in clinical information processing theories of PTSD, which can guide the development of effective treatments for trauma-related disorders.

## Data Availability

All anonymized data, analysis code, and syntax for reproducing the presented findings are available on OSF via this link (see Supplementary Material).
